# Supplementary material for: Easy-to-Operate Co-Flow Step Emulsification Device for High-Throughput Three-Dimensional Cell Culture
Source: Biosensors (Basel). 2022 May 18;12(5):350. doi: 10.3390/bios12050350 (PMC9138713; doi:10.3390/bios12050350)
Supplement: Supplementary file 1 [file biosensors-12-00350-s001.zip › biosensors-1722117-supplementary.pdf]

# Supporting Information

## Easy-to-Operate Co-flow Step Emulsification Device for High-Throughput Three-Dimensional Cell Culture

**Chunyang Wei [1], Chengzhuang Yu [2], Shanshan Li [1,2,3], Tiejun Li [1] *, Jiyu Meng [2], and Junwei Li [4,5,*]**

[1] Hebei Key Laboratory of Robotic Sensing and Human-robot Interactions, School of Mechanical Engineering, Hebei University of Technology, Tianjin 300132, China

[2] State Key Laboratory of Reliability and Intelligence of Electrical Equipment, Hebei University of Technology, Tianjin 300130, China

[3] Jiangsu Key Laboratory of Advanced Food Manufacturing Equipment and Technology, Wuxi 214122, China

[4] Institute of Biophysics, School of Health Sciences and Biomedical Engineering, Hebei University of Technology, Tianjin 300401, China

[5] Department of Electronics and Information Engineering, Hebei University of Technology, Langfang 065000, China

Corresponding authors:

Tiejun Li, li_tiejun@hebut.edu.cn;

Junwei Li, junwei_li@hebut.edu.cn

## Note S1. Fabrication of the step microstructure

The specific step microstructure was formed by fixing the PDMS replica tightly onto a clean glass substrate and drilling a hole directly above the straight shallow microchannel. Figure S1 shows the "alignment" between the bio-puncher and the straight microchannel; it could be finished in just a few seconds with the help of human eyes. Therefore, we did not need to make a step mold by the multilayer lithography technique. The combination of “single-layer photolithography” and the “punching operation” is suitable for the fabrication of SE microfluidic devices with step microstructures which are economical and easy to produce.

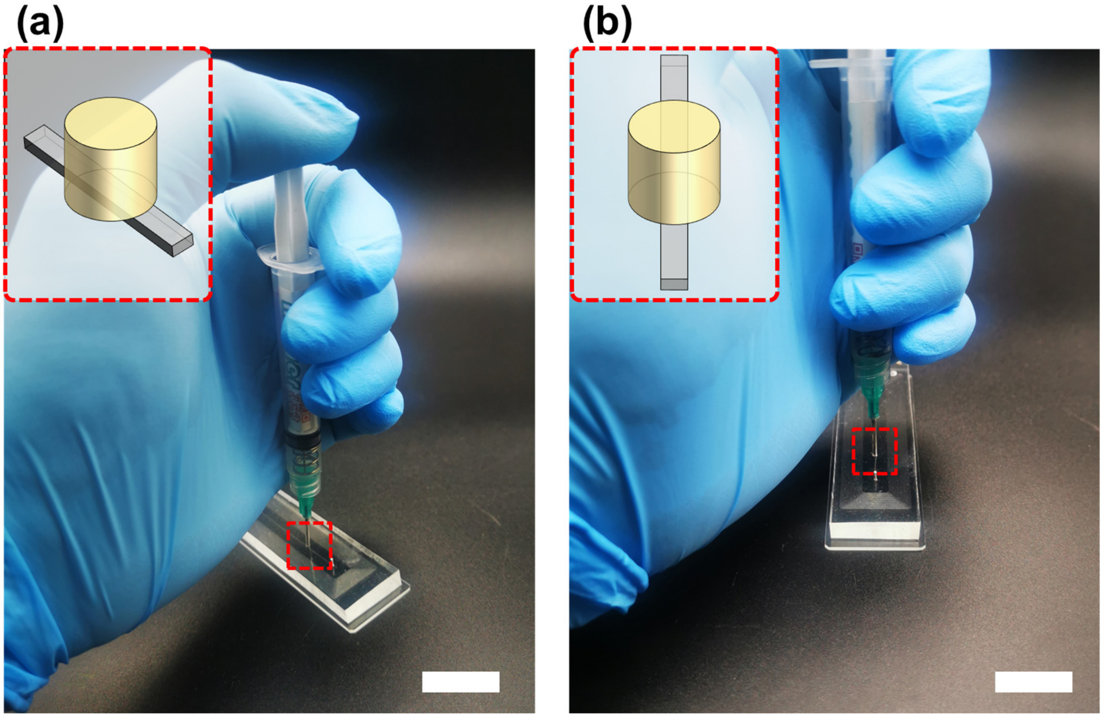


**Figure S1**. Fabrication of the step microstructure. The bio-puncher was placed vertically right on the microchannel. Scale bars: 2 cm.

## Note S2. A schematic diagram of our "punching method" and "multilayer lithography" for the fabrication of a step microstructure

Figure S2 shows the schematic diagram of our "punching method" and "multilayer lithography" [1,2] for the fabrication of a step microstructure. In our "punching method", a 4-inch silicon wafer was thoroughly cleaned before being used. Then, SU8-2075 negative photoresist (MicroChem, Westborough, MA, USA), 40 μm thick, was coated on the glass wafer (1), after which the wafer was covered with a mask (2) and exposed to ultraviolet radiation for SU8 crosslinking (3). After development (4), PDMS was cast on the mould and baked for 2 hours (5a) to obtain a PDMS replica (6a). Finally, the microchannels in the PDMS replica were cut with a bio-puncher to produce a step microstructure. The "multilayer lithography" method of obtaining a step microstructure involves more steps than our method, including photoresist coating (5b), mask alignment (6b), UV exposure (7b), and photoresist development (8b), PDMS casting (9b), and demoulding (10b); it is more complicated, expensive, and time-consuming.

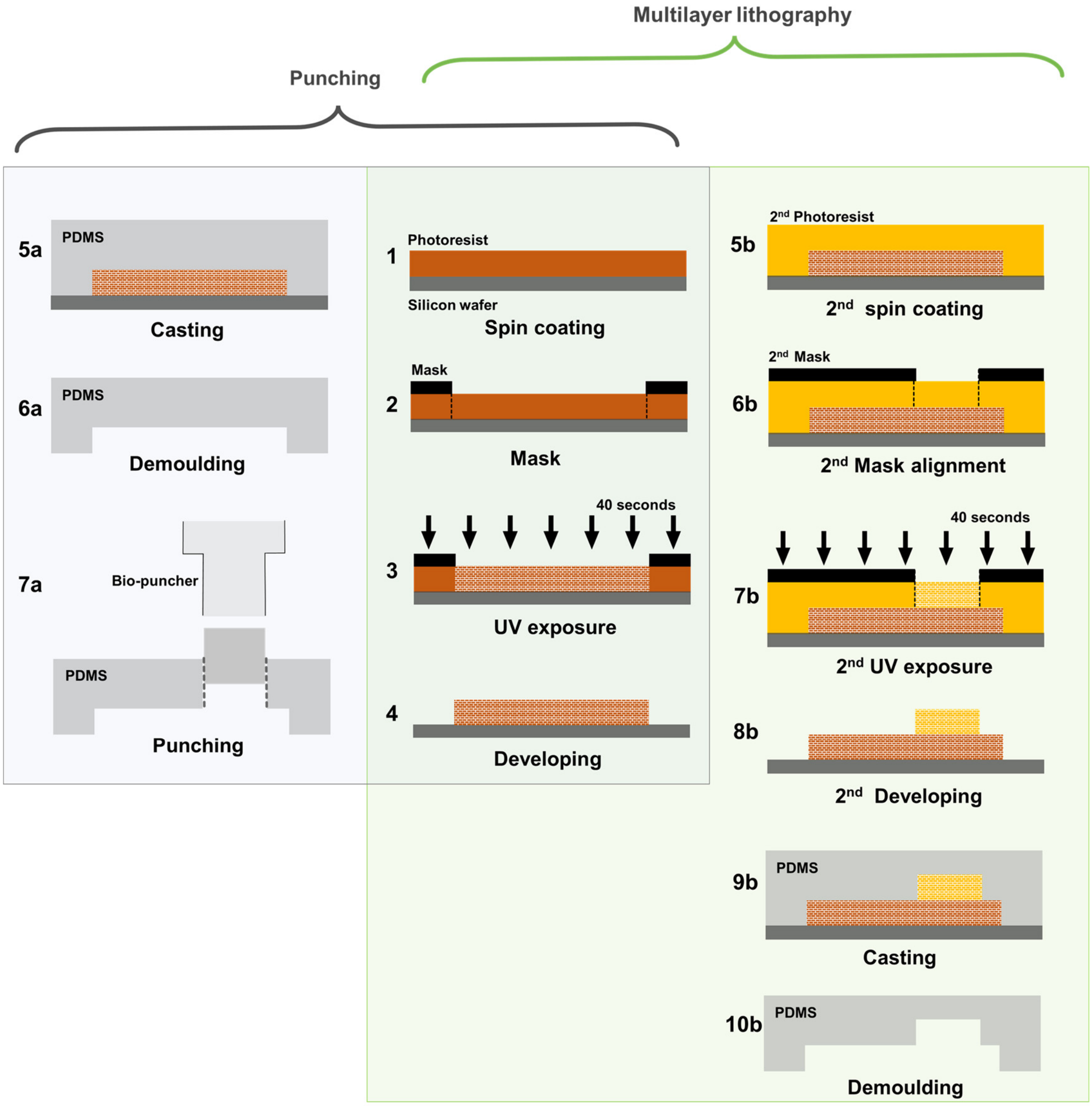


**Figure S2.** Schematic diagram of "punching" and "multilayer lithography" for obtaining a step microstructure. For both methods: 1: Coat a silicon wafer with photoresist; 2: Cover the wafer with a designed mask; 3: Expose the covered wafer to ultraviolet light; 4: Develop to form the mould of microchannels. For the "punching" method: 5a: Cast PDMS on the obtained mould; 6a: Demould to obtain a PDMS replica; 7a: Punch the shallow microchannel to obtain a step microstructure. For "multilayer lithography": 5b: Coat the first layer with photoresist; 6b: Cover the second layer of photoresist with a designed mask; 7b: Expose the covered wafer to ultraviolet light again; 8b: Develop to form the mould of microchannels. 9b: Cast PDMS on the obtained mould; 10b: Demould to obtain a PDMS replica.

## Note S3. The CFSE droplet generation based on the Rayleigh–Plateau instability

As shown in Figure S3, when oil (the outer-phase liquid) and cell medium (the inner-phase liquid) meet each other at the cross-junction, they can maintain a laminar flow because of their low Reynolds numbers. This state is called “co-flow” [3]. The co-flow occurs in the regions labelled “A” in the two-dimensional schematic of Plan-P3.

To generate a water-in-oil droplet, part of the co-flow segment must be separated from the body, meaning that the stable state must be broken. Here, we use the Rayleigh–Plateau instability to break the sample stream. As shown in Figure S3, an exquisite and precise step structure is well designed and fabricated to combine the narrow-and-low co-flow channel and the wide-and-high bulky storage reservoir. When the co-flow enters the cylindrical space from the shallow straight channel, the movement space is instantaneously expanded from a micron to a millimeter scale. Therefore, the co-flow will experience a sudden geometric change in the vertical and horizontal directions. A tongue-shaped precursor is formed and then detached from the body fluid (regions labelled “B” and “C”). Then, the separated precursor will form a spherical droplet (region labelled "D"). The corresponding video is shown in Movie S1.

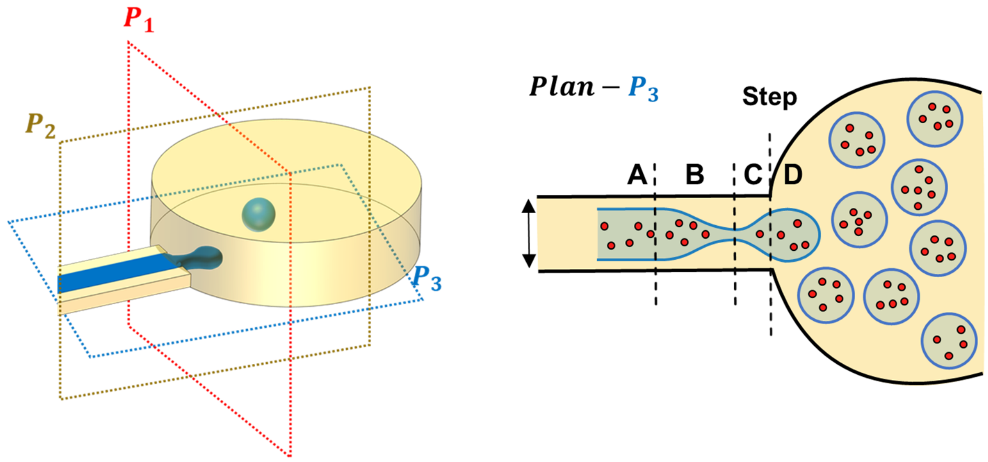


**Figure S3**. Demonstration of the co-flow step emulsification. The left subfigure is a three-dimensional diagram of droplet formation at the step structure. The right subfigure is a plan diagram of the tongue-shaped precursor.

## Note S4. Investigation of the thermal stability and chemical stability of the microfluidic CFSE device

The “thermal stability” of the CFSE device is affected by “cell medium temperature” and “ambient temperature”.

Investigation of “cell medium temperature”: We employed cell media of different temperatures (20, 25, 30, 35, and 40 °C) for five independent droplet generation experiments. (The ambient temperature was fixed at 25°C.) Figure S4a shows the droplet generation stability in the proposed device with cell media of different temperatures. Here, the flow rates of the inner phase and the outer phase were set at 1.28 and 0.49 μL/min, respectively. From the plot of the measured droplet diameter, the droplet sizes remained at 80.78 ± 2.54 μm, showing excellent stability (CV < 5%).

Investigation of “ambient temperature”: We carried out droplet generation experiments at different ambient temperatures (20, 25, 30, 35, and 40 °C). Figure S4b shows the droplet generation stability in the proposed device under different ambient temperatures. Here, the flow rates of the inner phase and the outer phase were set at 1.28 and 0.49 μL/min, respectively. From the plot of the measured droplet diameter, the droplet sizes remained at 82.16 ± 2.72 μm, showing excellent stability (CV < 5%).

Since the temperatures of most cell culture scenarios are in the range of 20 ~ 40 degrees, the

experiments above are sufficient to demonstrate the excellent thermal stability of the CFSE device.

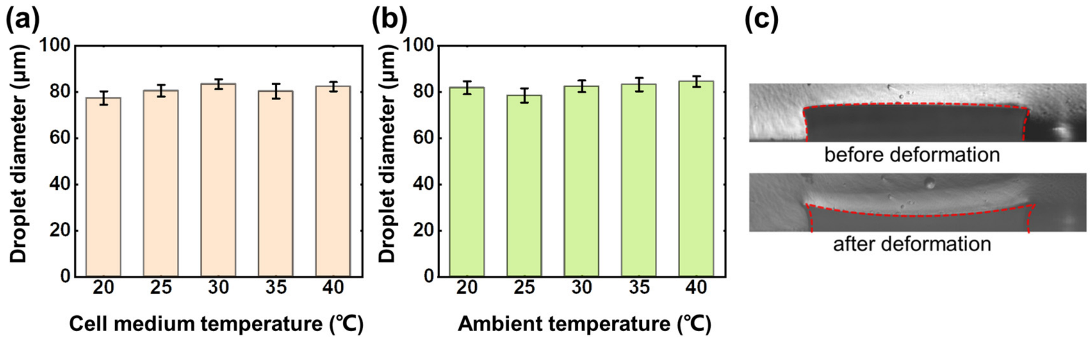


**Figure S4.** **(a)** Cell medium temperature-dependence of droplet diameter; **(b)** Ambient temperature-dependence of droplet diameter; **(c)** The cross-sections of the microchannel before and after PDMS deformation.

Investigations of the “chemical stability” of the CFSE device: The CFSE device consists of a replica and a glass substrate: the glass substrate has good chemical stability, while PDMS may be affected by organic solvents.

For most experiments, cell suspensions are aqueous and would not react with PDMS; thus, the CFSE device can be reused many times (at least 20 times for the saccharomyces cerevisiae cell culture experiments). For a few specific experiments which involve “organic solvents”, the organic solvent will penetrate the microchannel because of the porous nature of PDMS, resulting in geometric deformation on the roof of the microchannel (Figure S4c). This will eventually lead to multi-dispersed droplets or even the blockage of microchannels.

## Note S5. The CFSE device's capability to make droplets with custom sizes

Figure S5 shows the CFSE device's capability to make droplets with custom sizes. The droplet diameter is regulable by the fluid flow rates; thus, we can make diverse droplet chambers to achieve flexible cell encapsulation. Figure S5a shows droplet arrays with an average diameter of 62.62 μm; approximately four cells were encapsulated in each droplet. Figure S5b shows droplet arrays with an average diameter of 91.09 μm; approximately 25 cells were encapsulated in each droplet.

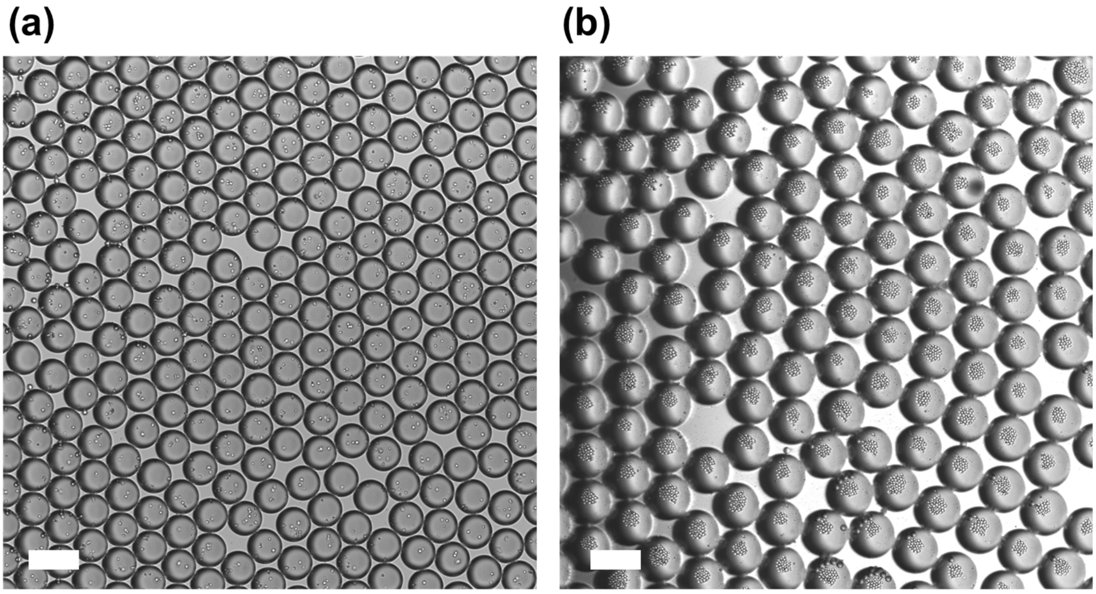


**Figure S5**. Saccharomyce cerevisiae cells encapsulated in different sized droplets. **(a)** $\Phi$ = 62.62 ± 1.95 μm, $Q_{inner\text{-}phase}$ = 1.05 μL/min, $Q_{outer\text{-}phase}$ = 0.41 μL/min; cell concentration = 1.38 × $10^7$ cells/mL; **(b)** $\Phi$ = 62.62 ± 1.95 μm, $Q_{inner\text{-}phase}$ = 1.43 μL/min, $Q_{outer\text{-}phase}$ = 0.56 μL/min; cell concentration = 8.27 × $10^7$ cells/mL. Scale bars: 100 μm.

## Note S6. Comparison of the CFSE platform and a commercial 3D culture platform

We compared the CFSE platform with a commercial 3D culture platform (12-60TR-Rods, Microtissues, USA). The proliferation rates and proliferation times of saccharomyces cerevisiae cells were investigated, and the corresponding data are shown in Figure S6. Statistical analysis demonstrated that our in-droplet culture mode had a similar proliferation rate and time to the commercial 3D culture platform. As the proposed CFSE device is more economical and easy to operate, it can serve as a promising and effective tool for cell research, including the study of metabolism, growth, differentiation, interactions, and other functions.

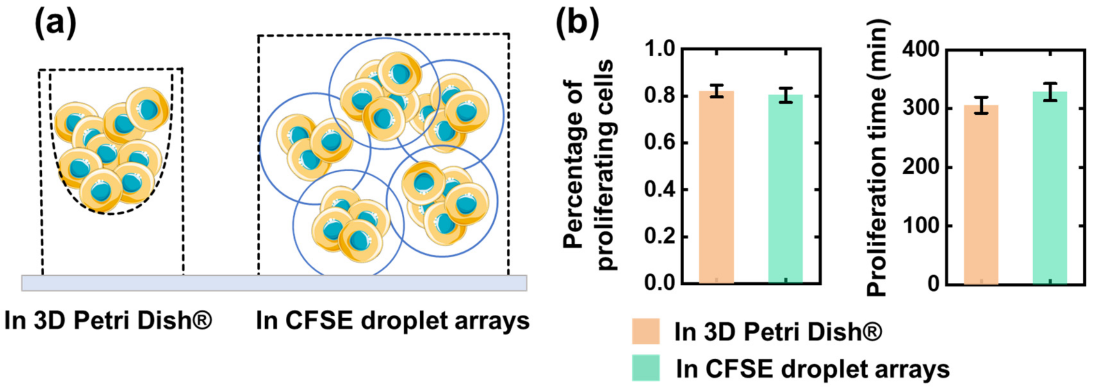


**Figure S6**. (a) Schematic diagram of in 3D Petri dish platform and in CFSE droplet array platform; **(b)** Comparison of results for proliferation rates and proliferation times.

**References:**

[1] Xia, Y.; Whitesides, G.M. Soft lithography. *Annu. Rev. Mater. Sci.* **1998**, *28*, 153–184.

[2] Whitesides, G. M.; Ostuni, E.; Takayama, S.; Jiang, X.; Ingber, D. E. Soft lithography in biology and biochemistry *Annu. Rev. Biomed. Eng.* **2001,** *3*, 335–373.

[3] Li, Z.; Leshansky, A.; Pismen, L.; Tabeling, P. Step-emulsification in a microfluidic device *Lab Chip* **2015,** *15* , 1023–1031.
